# Comparison of Continuous Thoracic Epidural Analgesia Versus Bilateral Erector Spinae Plane Block for Pain Management in Coronary Bypass Surgery

**DOI:** 10.7759/cureus.67149

**Published:** 2024-08-18

**Authors:** Vipul Sharma, Harika Atluri

**Affiliations:** 1 Anesthesiology, Dr. D. Y. Patil Medical College, Hospital and Research Centre, Dr. D. Y. Patil Vidyapeeth, Pune, IND

**Keywords:** ultrasound guided block, spirometry, visual analog scale, analgesia, thoracic epidural, erector spinae plane block

## Abstract

Background

Effective pain control is vital for patients undergoing heart surgery. Utilizing a multimodal approach to analgesia is essential, as poor pain management can result in hemodynamic and systemic complications. This study aimed to compare perioperative pain management techniques in patients undergoing coronary artery bypass grafting (CABG), specifically evaluating continuous thoracic epidural analgesia and ultrasound-guided bilateral erector spinae block.

Methods

This randomized comparative study was conducted at a tertiary care centre over a period of six months, with approval from the institute's ethics committee. A total of 24 patients undergoing CABG under general anesthesia participated in the study. They were randomly assigned to either the continuous thoracic epidural analgesia (TEA) group (Group A) or the ultrasound-guided bilateral erector spinae plane (ESP) block group (Group B) using a simple randomization method. The study assessed intraoperative intravenous opioid requirements for maintaining stable hemodynamics, as well as postoperative resting and coughing Visual Analog Scale (VAS) scores and peak inspiratory spirometry.

Results

Twelve patients from each group completed the study, with comparable demographics (age, gender). Both groups exhibited similar resting and coughing VAS scores at 0, 3, 6, and 12 hours postoperatively (p > 0.05). However, at 24, 36, and 48 hours, Group A had significantly higher VAS scores compared to Group B (p < 0.05). Group A maintained an overall mean VAS score of 4 or less during rest and coughing. Peak inspiratory spirometry results were consistent between both groups (p > 0.05).

Conclusion

The ultrasound-guided bilateral erector spinae block provided pain control comparable to thoracic epidural analgesia, making it a viable alternative for perioperative pain management. This is particularly beneficial for CABG patients where early postoperative anticoagulant therapy is crucial for graft patency. Effective pain management also contributes to faster recovery in coronary artery bypass grafting.

## Introduction

Coronary artery bypass grafting (CABG) is the most commonly performed major surgery for patients with coronary artery disease. In this procedure, atheromatous obstructions in the coronary arteries are surgically bypassed using harvested venous or arterial conduits. Postoperative pain following CABG often escalates to moderate or severe levels due to factors such as the insertion of chest tubes, sternotomy, and the dissection of the internal mammary artery. Inadequate pain management can lead to complications like lung atelectasis, increased oxygen consumption, and hemodynamic instability [1].

Historically, intravenous opioids and non-steroidal anti-inflammatory drugs (NSAIDs) have been the mainstay for managing acute pain after cardiac surgeries. While opioids effectively control pain and aid in sedation, they also carry risks like drowsiness, respiratory depression, and cardiac depression. Consequently, there is a growing trend towards minimizing opioid usage by integrating non-opioid analgesics and targeted local blocks [2].

The thoracic epidural blockade has traditionally been considered the gold standard for neuraxial anesthesia in managing sternotomy pain. However, it poses significant risks, including the potential for epidural hematoma leading to paraplegia, especially with the use of heparin during cardiac surgeries and early initiation of postoperative anticoagulation therapy [3]. While paravertebral blockade provides pain relief comparable to thoracic epidural anesthesia, its adoption is limited due to potential complications such as vascular injury or pneumothorax [4].

In contrast, the erector spinae plane (ESP) block has emerged as a viable alternative. This technique offers a simpler and safer method compared to traditional paravertebral and thoracic epidural blocks [5]. Introduced by Forero et al. in 2016 to address thoracic neuropathic pain and pain following mastectomy, ESP block involves administering local anesthetics above the transverse process and beneath the erector spinae muscle [6].

Cardiac anesthesia has traditionally relied on high doses of opioids to maintain hemodynamic stability and provide adequate analgesia. However, patients often experience unpleasant side effects such as urinary retention, nausea, vomiting, constipation, respiratory depression, and cardiac depression, which can hinder recovery [6]. Even with the extensive use of regional nerve blocks in multimodal analgesia, cardiac surgeons frequently employ heparinization, a procedure that increases the risk of major bleeding or hematoma, to administer techniques like thoracic epidural analgesia and paravertebral blocks [7]. Thus, another option could be superficial interfacial plane blocks guided by ultrasound [8].

ESP block is frequently used in various surgeries and is recognized for alleviating postoperative pain and promoting better recovery outcomes [9,10]. This study aims to evaluate the effectiveness of continuous thoracic epidural analgesia versus ultrasound-guided single-shot bilateral ESP block in controlling perioperative pain for patients undergoing CABG. Additionally, the study examines the impact of these pain control methods on incentive spirometry results in the intensive care unit.

## Materials and methods

This randomized comparative study was conducted in the Department of Cardiothoracic and Vascular Surgery, Dr. D. Y. Patil Medical College, Hospital and Research Centre, a tertiary hospital in Pune. The study spanned a period of six months (January to June 2024), with four months dedicated to data collection, and another two months for data processing and analysis. Ethical clearance (approval no: approval IESC/FP/80/2023) was obtained from the institute's ethics sub-committee before the study commenced. Each participant underwent a pre-anesthetic evaluation and relevant laboratory investigations. Informed written consent was obtained from all participating patients.

Patients from either gender aged between 35 and 75 years, classified according to the American Society of Anesthesiologists (ASA) as ASA II or ASA III, with a left ventricular ejection fraction (LVEF) greater than 35%, and undergoing CABG were eligible for inclusion. Additionally, participants needed to be capable of understanding the pain scale and sedation score. Exclusion criteria included patients from either gender with an ASA physical status of IV or higher, those unwilling to participate, or individuals with a history of hypersensitivity to study drugs such as morphine and bupivacaine. Patients were also excluded if they had epilepsy, liver or renal disease, chronic pain syndrome, psychiatric disorders, an LVEF below 35%, vertebral column anomalies, or did not meet the specified inclusion criteria.

Sample size calculation

The study comprised two groups: the continuous thoracic epidural analgesia (TEA) group and the ultrasonography (USG)-guided bilateral ESP block group. To detect a significant difference in mean visual analog scale (VAS) scores between the two groups with 95% confidence and 80% power, a minimum total sample size of 24 participants was required, with 12 participants per group. The sample size calculation was performed using IBM SPSS Statistics for Windows, Version 27 (IBM Corp., Armonk, NY).

Study procedure

After obtaining consent from patients from either gender and their relatives, a comprehensive pre-anesthetic assessment, including history taking and clinical examination, was conducted. Vital parameters such as height, pulse rate, blood pressure, respiratory rate, and any other clinical findings were recorded. Routine investigations, including hemoglobin levels and urine analysis, were performed for all patients. Additional tests, such as blood sugar, electrocardiogram (ECG), and chest X-ray, were performed as deemed necessary.

The study included a sample size of 24 patients, with 12 assigned to each of the two groups, namely group A and group B. Patients in group A received continuous TEA, while those in group B underwent a USG-guided bilateral ESP block. Random allocation of patients to either group ensured unbiased distribution.

Protocol

The patients were re-assessed preoperatively, and it was ensured that heparin, aspirin, and clopidogrel were withheld appropriately, and the international normalized ratio (INR) was confirmed to be less than 1.5. The patient received 0.25 mg of alprazolam the night before and on the morning of the surgery. Additionally, an 18/16-gauge(G) IV line was established for administering fluids and medications, and 1 mg of tranexamic acid was given via Intravenous infusion. An 18G Tuohy epidural needle, a loss of resistance syringe, epidural catheters, and emergency equipment and medications were prepared. In the operation theater (OT), all routine monitors (ECG, non-invasive blood pressure, and pulse oximeter) were attached for both groups.

In group A, with the patient in a sitting position, an 18G epidural needle was inserted at the T4-T5 or T5-T6 intervertebral space using the loss of resistance or hanging drop method. The epidural catheter was advanced 3 cm into the epidural space. A bolus of 10 ml of 0.25% bupivacaine and 3 mg of morphine was administered in 2 ml increments over 30 minutes.

In group B, the USG-guided single-shot ESP block was meticulously performed following strict aseptic protocols. Utilizing a linear ultrasonic transducer with a high frequency of 12 MHz, we positioned it longitudinally, three centimeters lateral to the T6 spinous process, corresponding to the T5 transverse process. By visualizing above the hyperechoic transverse process, we identified three muscles: the upper trapezius, middle rhomboids major, and lower erector spinae. Local injection of 2% lignocaine was administered at the needle insertion site. Following that, 5 ml of normal saline was injected, and after observing hydro dissection beneath the muscle plane, an 18G Tuohy needle was inserted in a caudal-cephalad trajectory until the tip of the needle reached the erector spinae muscle. A single-shot injection of 10 ml of 0.25% bupivacaine and 3 mg of morphine was administered equally on each side.

The anesthesia technique was identical in both groups, starting with the administration of midazolam at 0.02 mg/kg, fentanyl at 2 mcg/kg, and propofol at 2 mg/kg for premedication and induction respectively, followed by muscle relaxation with vecuronium at 0.1 mg/kg. After gentle laryngoscopy, an appropriately sized endotracheal tube was inserted and secured after ensuring bilateral equal air entry. Central venous pressure (CVP) and arterial lines were then established. Any drop in blood pressure was managed with a single dose of 50 to 100 mcg of phenylephrine, repeated as necessary.

Intraoperative hemodynamic assessment (blood pressure, heart rate, and saturation) was done at various stages, including skin incision, sternotomy, and every 15 minutes thereafter.

One hour after epidural catheter insertion, Heparin was administered at a rate of 2-4 mg/kg body weight, followed by reversal with Protamine sulfate. Extubation occurred when the patient was hemodynamically stable, without ongoing blood loss, and off inotropes. Epidural catheters were removed on postoperative day 1.

Breakthrough pain was managed with injection (Inj.) paracetamol 1 g and Inj. tramadol 50 mg. Postoperative monitoring included blood pressure, heart rate, and saturation, with pain assessment. Other side effects such as nausea, vomiting, dizziness, and confusion were also noted.

VAS was used to assess pain levels, with evaluations at various intervals post-extubation both during rest and coughing. Spirometry was performed to assess peak inspiratory flow rate. A resting VAS score of 4 or above was considered indicative of breakthrough pain [11]. Intravenous paracetamol 1 g was administered every six hours to all patients, with additional rescue analgesia with intravenous tramadol provided as needed.

Statistical methods

The data was coded and entered into a Microsoft Excel 2019 (Microsoft Corp., Redmond, WA) spreadsheet. Statistical analysis was conducted using the software IBM SPSS Statistics for Windows, Version 27 (IBM Corp., Armonk, NY). Descriptive statistics included calculating means and standard deviations. The Chi-squared test was used for categorical data comparison, while the independent t-test was employed for continuous variables. A p-value of <0.05 was considered significant.

## Results

**Figure 1:**
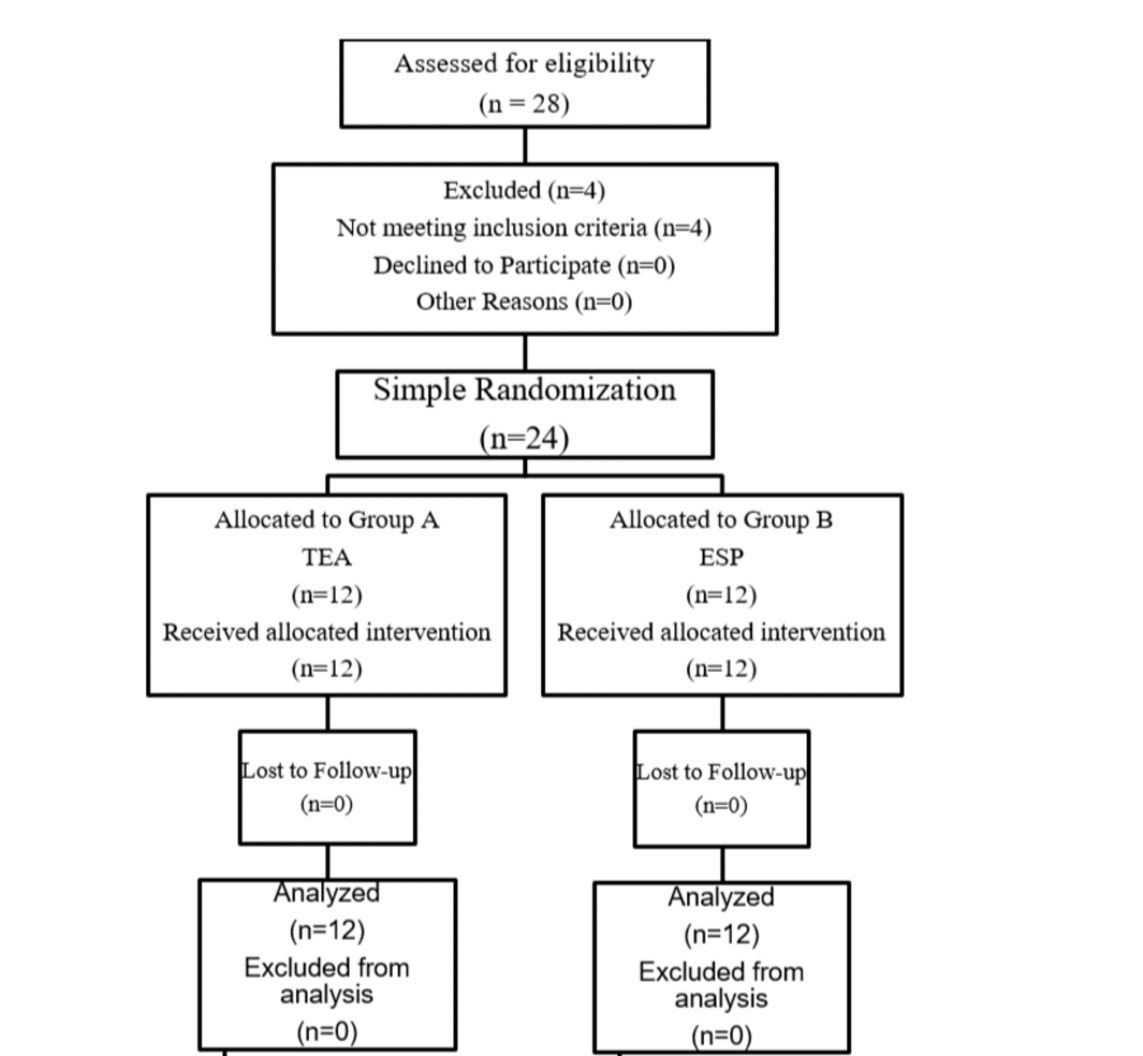
CONSORT chart of simple randomization. TEA: thoracic epidural analgesia, ESP: erector spinae plane, CONSORT: Consolidated Standards of Reporting Trials.

In this randomized comparative study, 28 patients were initially assessed, with 4 excluded due to not meeting the inclusion criteria. The remaining 24 patients were randomly assigned to two groups: Group A (TEA) and Group B (ESP), each consisting of 12 participants. All patients completed the study and were included in the analysis (Figure 1).

The study was completed by a total of 12 patients in each group. Both groups showed similarities in terms of age and gender distribution (Table 1). Group A had a mean age of 50.12 years with 58.33% males and 41.67% females. Group B had a mean age of 45 years with 66.67% males and 33.33% females. Key clinical measures included intraoperative fentanyl dosage, ventilator duration, and intensive care unit (ICU) duration, with no significant differences between groups except for a notably higher ICU duration in Group A compared to Group B.

**Table 1:** Demographic distribution of the study population. ICU: intensive care unit.

Variables	Group A	Group B	p-value
Age (years)	50.12±15.21	45±19.43	0.36
Sex			
Male	7 (58.33%)	8 (66.67%)	Not applicable
Female	5 (41.67%)	4 (33.33%)	Not applicable
Intraoperative fentanyl (mcg)	334±80.90	366.2±104.34	0.25
Ventilator duration (min)	296±51.98	296.6±54.66	0.84
ICU duration (min)	3848±964.96	3274±1206.32	0.07

The analysis of VAS scores at rest and during coughing (Tables 2, 3) revealed significant differences between the two groups at later time intervals. While the initial VAS scores were comparable, Group A consistently showed higher pain scores both at rest and during coughing from 24 hours onward. These findings suggest that patients in Group B, who received the ultrasound-guided bilateral ESP block, experienced better pain control in the postoperative period compared to those in Group A, who received continuous TEA. Despite these findings in the later hours, the overall mean VAS score in Group A remained ≤4 both at rest and during coughing.

**Table 2:** Distribution of VAS (rest) at different time intervals in the study population. Data are presented as mean ± SD (mean ± 95% CI) for VAS scores at rest measured at specific intervals between 0 and 48 hours for Group A and Group B (n=12 each). (*) denote p-values with statistically significant differences between the two groups, where p-values are less than 0.05. VAS: Visual Analog Scale.

Time (h)	Group A (n=12)	Group B (n=12)	p-value
	Mean ± SD (95% CI)	Mean ± SD (95% CI)	
0	1.58 ± 1.06 (1.58 ± 0.61)	1.06 ± 0.94 (1.06 ± 0.54)	
3	1.54 ± 0.66 (1.54 ± 0.38)	1.50 ± 1.00 (1.50 ± 0.70)	0.65
6	1.62 ± 0.68 (1.62 ± 0.35)	1.66 ± 1.34 (1.66 ± 0.61)	1.00
12	1.90 ± 0.92 (1.90 ± 0.53)	1.68 ± 1.32 (1.68 ± 0.71)	0.48
24	2.06 ± 0.62 (2.06 ± 0.36)	1.46 ± 0.86 (1.46 ± 0.50)	0.007*
36	2.26 ± 1.04 (2.26 ± 0.60)	1.07 ± 0.83 (1.07 ± 0.41)	0.0003*
48	2.00 ± 1.34 (2.00 ± 0.72)	0.80 ± 0.62 (0.80 ± 0.36)	0.0001*

**Table 3:** Distribution of VAS (cough) at different time intervals in the study population. Data are presented as mean ± SD (mean ± 95% CI) for VAS scores during cough measured at specific intervals between 0 and 48 hours for Group A and Group B (n=12 each). (*) denote p-values with statistically significant differences between the two groups, where p-values are less than 0.05. VAS: Visual Analog Scale.

Time (h)	Group A (n=25)	Group B (n=25)	p-value
	Mean ± SD (95% CI)	Mean ± SD (95% CI)	
0	2.18 ± 1.22 (2.18 ± 0.48)	1.89 ± 1.34 (1.89 ± 0.71)	
3	2.38 ± 0.78 (2.38 ± 0.31)	2.46 ± 0.94 (2.46 ± 0.35)	0.78
6	2.54 ± 0.86 (2.54 ± 0.33)	2.70 ± 1.28 (2.70 ± 0.50)	0.82
12	2.78 ± 1.12 (2.78 ± 0.44)	2.50 ± 1.48 (2.50 ± 0.58)	0.38
24	3.06 ± 0.72 (3.06 ± 0.28)	2.37 ± 1.08 (2.37 ± 0.47)	0.007*
36	2.97 ± 1.23 (2.97 ± 0.48)	1.90 ± 1.07 (1.90 ± 0.42)	0.0004*
48	2.74 ± 1.34 (2.74 ± 0.71)	1.37 ± 0.73 (1.37 ± 0.27)	0.0001*

The spirometry assessment in the study population at various time intervals postoperatively gives the following analysis: (a) Early time points (3h, 6h, 12h): Group A consistently demonstrates higher peak inspiratory flow compared to Group B. However, these differences are not statistically significant, as indicated by p-values slightly above the threshold; (b) Later time points (24h, 36h, 48h): As time progresses, the differences in peak inspiratory flow between the two groups diminish and remain statistically insignificant. Both groups eventually achieve similar levels of inspiratory flow; (c) Overall trend: Despite the initial trend of higher inspiratory flow in Group A, this difference does not reach statistical significance. Over time, both groups exhibit comparable inspiratory flow, indicating that both analgesic techniques are equally effective in maintaining respiratory function postoperatively (Table 4).

**Table 4:** Distribution of peak inspiratory flow (spirometry) in the study population at different time intervals. Spirometry measurements (mean ± SD and 95% CI) at various time points for Group A and Group B, with n=12 for each group. A p-value less than 0.05 indicates statistically significant differences between groups. However, the data provided above does not show such significant differences.

Time (h)	Group A (n=12)	Group B (n=12)	p-value
	Mean ± SD (95% CI)	Mean ± SD (95% CI)	
3	752 ± 128.92 (752 ± 73.54)	676 ± 152.78 (676 ± 86.43)	0.08
6	818 ± 108.72 (818 ± 61.07)	746 ± 174.76 (746 ± 98.76)	0.07
12	856 ± 95.05 (856 ± 53.55)	782 ± 182.72 (782 ± 103.31)	0.07
24	856 ± 112.58 (856 ± 63.44)	884 ± 92 (884 ± 52.28)	0.45
36	872 ± 134.92 (872 ± 75.62)	908 ± 112.22 (908 ± 63.92)	0.32
48	886 ± 95.05 (886 ± 53.55)	908 ± 112.24 (908 ± 63.40)	0.56

## Discussion

Effective pain management during the perioperative period is crucial for patients undergoing heart surgery. The pain associated with cardiac surgery, including procedures such as sternotomy, sternal retraction, internal mammary artery harvesting, and chest tube insertions, is typically moderate to severe. Inadequate pain control can lead to hemodynamic disturbances and systemic complications, such as lung issues (atelectasis, pneumonia, and bronchial secretion stagnation), cardiovascular problems (increased oxygen consumption and tachycardia), muscle weakness, and an increased neurohormonal response [12]. The ASA task force recommends multimodal approaches for acute postoperative pain management, including local analgesia and intravenous (IV) and oral analgesics [13]. Parenteral analgesics, such as opioids, paracetamol, and nonsteroidal anti-inflammatory drugs, are commonly used; however, opioids can cause side effects like nausea, vomiting, itching, and respiratory depression. Neuraxial techniques, such as TEA, have been well-documented for their effectiveness in managing postoperative pain after heart surgery, resulting in better outcomes. Paravertebral blocks (PVBs) have shown similar effectiveness to TEA, particularly in minimally invasive heart surgery [14].

The safety of neuraxial anesthesia (NA) methods in cardiac surgery patients is a critical concern, particularly for those on long-term antiplatelet medications, undergoing intraoperative systemic anticoagulation, or experiencing coagulopathy due to cardiopulmonary bypass. The incidence of epidural hematoma in cardiac surgery is not well-defined [5]. However, the estimated risk of an epidural hematoma with TEA is approximately one in 12,000, and the risk of catheter-related epidural hematoma is about one in 5,493 [15]. Despite these risks, the use of NA analgesia in cardiac surgery has increased with the adoption of less invasive procedures. Recent studies have highlighted the therapeutic benefits of intrapleural, paravertebral, and intercostal blocks compared to traditional epidural techniques.

Ho et al. demonstrated the effectiveness of an ESP block using a continuous catheter for pain relief in patients with multiple unilateral rib fractures [16]. They proposed that the cephalocaudal spread of the local anesthetic (LA) is due to its proximity to the costotransverse foramina, where the dorsal and ventral rami of the thoracic spinal nerves originate. The thoracolumbar fascia, which extends from the thorax and abdomen to connect with the nuchal fascia of the neck, further facilitates the spread of the LA. In their study, pain relief was effectively achieved, with VAS scores remaining below 4 for 48 hours post-extubation using a continuous catheter. Foreroet al.* *reported the successful use of the ESP block as a rescue analgesic technique in thoracotomy after a failed epidural [6]. TEA and paravertebral blocks (PVB) are frequently chosen as the primary regional analgesic methods for pain management in thoracic surgeries [17].

Bonvicini et al. reported the use of bilateral ultrasound-guided ESP block in breast cancer reconstructive surgery, suggesting it as a viable alternative to PVB and TEA [18]. The sonoanatomy is easily identifiable, with no critical structures at risk of needle injury. The authors achieved a 100% success rate with the USG ESP block, with no failed blocks. Complications related to epidural analgesia in cardiac surgery have been documented by Hoet al.* *[16]. In their study, no complications were reported in any of the groups. Therefore, the researchers concluded that the ESP block is a highly promising alternative to TEA for providing perioperative analgesia.

This study's findings revealed that both techniques were effective immediately post-surgery, but the ESP block provided superior pain relief at 24, 36, and 48 hours postoperatively. The ESP block demonstrated comparable pain scores to TEA during rest and coughing, with the added benefits of simplicity, safety, and fewer complications. It consistently maintained a mean VAS score of 4 or less, indicating effective pain management. Additionally, peak inspiratory spirometry results were similar for both groups, suggesting the ESP block does not adversely affect pulmonary function, which is crucial for cardiac surgery recovery. The lack of significant side effects and complications further supports the ESP block's potential to enhance postoperative recovery and patient comfort.

Limitations

The study has a few limitations. Firstly, with only 12 patients in each group, the small sample size limits the generalizability of the findings. Conducting the study at a single center further restricts its applicability, suggesting that multi-center studies would offer better external validity. Additionally, the lack of blinding may introduce bias, while variations in surgical and postoperative care highlight the need for standardization.

Furthermore, the study predominantly addresses immediate postoperative pain relief, without considering long-term outcomes such as chronic pain, functional recovery, or quality of life. Unmeasured factors, such as baseline pain thresholds and psychological influences, could potentially affect the results. Lastly, the study's homogenous population and the exclusion of certain patient groups might limit the relevance of the findings to a broader patient demographic.

## Conclusions

The ultrasound-guided bilateral erector spinae plane (ESP) block appears to be a promising and safer alternative for continuous thoracic epidural analgesia (TEA) in patients undergoing coronary artery bypass grafting (CABG), particularly when anticoagulants are involved. It delivers effective pain management with fewer complications and reduced opioid consumption. However, additional research with larger, multicentric studies is necessary to verify its wider applicability and to potentially standardize its use.
